# Amphetamine use and mental health difficulties across adolescence and young adulthood: An integrative data analysis of four Australasian cohort studies

**DOI:** 10.1111/add.70033

**Published:** 2025-03-15

**Authors:** Christopher J. Greenwood, James Foulds, Rebecca McKetin, Stephanie R. Aarsman, Delyse Hutchinson, Jessica Kerr, Jessica A. Heerde, John W. Toumbourou, Joseph M. Boden, Tim Slade, Yvonne Bonomo, Primrose Letcher, Craig A. Olsson

**Affiliations:** ^1^ SEED Lifespan Strategic Research Centre, School of Psychology, Faculty of Health Deakin University Geelong Australia; ^2^ Centre for Adolescent Health Murdoch Children's Research Institute Melbourne Australia; ^3^ Department of Paediatrics, Royal Children's Hospital The University of Melbourne Australia; ^4^ Department of Psychological Medicine The University of Otago Christchurch New Zealand; ^5^ National Drug and Alcohol Research Centre University of New South Wales Sydney Australia; ^6^ Department of Social Work The University of Melbourne Melbourne Australia; ^7^ School of Population Health Curtin University Perth Australia; ^8^ The Matilda Centre for Research in Mental Health and Substance Use The University of Syndey Sydney Australia; ^9^ Department of General Practice, Faculty of Medicine, Dentistry and Health Sciences The University of Melbourne Melbourne Australia; ^10^ St Vincent's Health, Department of Addiction Medicine Melbourne Australia

**Keywords:** adolescent, amphetamine, causal, cohort, longitudinal, mental health, young adult

## Abstract

**Background and aims:**

The use of amphetamines (including amphetamine and methamphetamine) has been consistently associated with mental health difficulties; however, the direction of potential causal relationships has not yet been established. This study aimed to assess the direction relationships between illicit amphetamine use and mental health difficulties across adolescence and young adulthood.

**Design:**

Observational study of four population‐level cohorts participating in the Monitoring Illicit Substance Use (MISUse) Consortium.

**Setting:**

Australia and New Zealand.

**Participants:**

A total of 7527 participants (51% female) were used: Christchurch Health and Development Study (*n* = 1056), Australian Temperament Project (*n* = 1644), Victorian Adolescent Health Cohort Study (*n* = 1943) and International Youth Development Study (*n* = 2884).

**Measurements:**

Assessments were used to derive binary indicators of amphetamine use (≥monthly) and mental health difficulties during both adolescence (age 10–17 years) and young adulthood (age 18–30 years).

**Findings:**

Associations were estimated as Risk Ratios (RRs) with 95% confidence internals (CIs) using G‐computation procedures, while accounting for 15 potential confounding factors and interactions between exposure and both study cohort and participant sex. The risk of mental health difficulties in young adulthood was 21% greater (RR = 1.21, 95% CI = 1.04, 1.41) for those who reported monthly or more frequent amphetamine use in adolescence. The risk of monthly or higher amphetamine use in young adulthood was 19% greater (RR = 1.19, 95% CI = 0.99, 1.45) in those who reported mental health difficulties in adolescence. There was also some evidence to suggest that in males the strongest association was from amphetamine use to mental health difficulties (RR = 1.24, 95% CI = 0.95, 1.60), while in females the strongest association was from mental health difficulties to amphetamine use (RR = 1.33, 95% CI = 0.99, 1.78).

**Conclusions:**

There appears to be a bidirectional association between monthly or more frequent amphetamine use and mental health difficulties from adolescence to young adulthood.

## INTRODUCTION

The use of illicit amphetamines, including both amphetamine and methamphetamine, is associated with significant harms [1]. Globally, although approximately 34.2 million people age 15 to 64 years use amphetamines, it remains relatively uncommon at the population level (<1%). Amphetamine and methamphetamine use is most prevalent in North America (2.0%) followed by the Oceanic region (1.3%) [2]. Existing studies suggest amphetamine use is generally most prevalent during the transitional periods of adolescence (approximately age, 10–17 years) and young adulthood (approximately age 18–30 years) [3]. Further, it has been estimated that amphetamine dependence may affect as many as 7.4 million people globally, with Australasia having the highest estimated prevalence of disorder in the world [1].

Comorbid mental health problems are common among people who use amphetamines [4]. Extensive evidence supports the emergence of negative emotional states (eg, dysphoria, anxiety and irritability) through biological pathways as integral to the processes of addiction [5]. Further, recent meta‐analytic evidence suggests that the likelihood of mental health difficulties, such as depression, is increased in individuals reporting amphetamine use/disorder [6, 7]. Specifically, meta‐analytic estimates suggested a 30% increase in the odds of depression (k = 6, adjusted OR = 1.3) [6], with associations somewhat stronger in cross‐sectional (k = 4; adjusted OR = 1.66) designs compared to longitudinal designs (k = 2; adjusted OR = 1.18) [7].

Yet inferences about the direction of causality are limited because of a confluence of factors, namely a reliance on cross‐sectional study designs, non‐representative samples and non‐systematic control for confounding variables. Population‐based, cohort studies can address some of these limitations by assessing the temporal ordering of the relationship between amphetamine use and mental health symptomatology and more systematically accounting for key confounders in this relationship. For example, evidence from one Australian cohort study has suggested an association between adolescent amphetamine use and young adult depression and anxiety, albeit with a limited confounding set (ie, participant sex and cannabis use, and parental smoking and divorce/separation) [8]. Likewise, evidence from another Australian cohort study found an unadjusted association between adolescent symptoms of anxiety and depression and lifetime amphetamine use in young adulthood, albeit the effects were no longer evident after adjustment for several other contemporaneous risk factors [9]. Further, in a Canadian sample, prospective associations between late adolescent amphetamine use and elevated depressive symptoms 1‐year later were identified after adjusting for the contemporaneous use of other substances and numerous pre‐existing individual and contextual characteristics [10].

Relatedly, a further issue impacting knowledge to date is the low prevalence of amphetamine use limiting analytic power. This limitation can be addressed by bringing together multiple cohort studies through data harmonisation and integrative data analysis, which increases both the overall number of participants and number of participants reporting amphetamine use [11, 12, 13]. Such efforts are essential to understanding the direction of causation between amphetamine use and mental health difficulties, and in doing so, inform the development of social policy capable of effective intervention.

Here, we use data drawn from the Monitoring Illicit Substance Use (MISUse Consortium) [14], which brings together four of the longest running of population studies of health and development in Australia and New Zealand, to examine the direction of potential causal relationships between amphetamine use and mental health difficulties across adolescence and young adulthood. Specifically, we examine evidence for a causal relationship between (1) adolescent amphetamine use and young adult mental health difficulties; and (2) adolescent mental health difficulties and young adult amphetamine use. Further, in line with current recommendations [15] and evidence of pre‐clinical and clinical sex differences in methamphetamine use disorder and related health outcomes [16], we examined whether identified relationships differ across male and female participants.

## METHODS

### Participants

The MISUse Consortium includes four cohort studies (see Table 1 for a brief overview and the protocol for more information [14]): the Christchurch Health and Development Study [17] (CHDS) (established 1977, Christchurch, New Zealand; *n* = 1265); the Australian Temperament Project [18] (ATP) (established 1983, Victoria, Australia; *n* = 2443); the Victorian Adolescent Health Cohort Study [19] (VAHCS) (established 1992, Victoria, Australia; *n* = 2032); and the International Youth Development Study [20] (IYDS) (established 2002, Victoria, Australia; *n* = 2884). Material S1 provides a detailed description of the included cohort studies, for which all participants were unique to each cohort. All participants provided written informed consent after receiving a complete description of the study. The MISUse Consortium has been approved by the Royal Children's Hospital (Melbourne) Human Research Ethics Committee (HREC reference number 84060). To be included in the current study, participants were required to have provided responses to amphetamine use or mental health difficulty items in at least one wave of data during adolescence (age, 10–17 years) or young adulthood age (age, 18–30 years).

**TABLE 1:** Characteristics of included cohort studies.

Region	CHDS	ATP	VAHCS	IYDS
Location	Christchurch, New Zealand	Victoria, Australia	Victoria, Australia	Victoria, Australia
Year of recruitment	1977	1983	1992	2002
Age at recruitment	Birth	4–8 months	14 y	11, 13, 15 y
No. invited	1310	3000^a^	2032	3926
No. who entered the study	1265	2443	1943	2884
No. of waves	24	16	11	10
No. of adolescent (age 10–17 y) waves^b^	1	3	5	5
No. of young adult (age 18–30 y) waves^b^	3	3	3	4
Year of last wave	2017–2019	2014	2019–2021	2018

Abbreviations: ATP, Australian Temperament Project; CHDS, Christchurch Health and Development Study; IYDS, International Youth and Development Study; VAHCS, Victorian Adolescent Health Cohort Study.

^a^ Approximate number.

^b^ Waves used in the current study.

### Measures

#### Amphetamine use and mental health difficulties

Across cohorts, assessments of amphetamine use (excluding the non‐prescribed use of prescription amphetamines) and mental health difficulties across both adolescence and young adulthood varied, although sufficient commonalities existed to enable data harmonisation (Material S2). Further, similar approaches to harmonising substance use and mental health indicators have been made in research from other consortia including these cohorts (eg, the Cannabis Cohort Research Consortium [11] and the Intergenerational Cohort Consortium [21]).

Within cohorts, amphetamine use was assessed using either past year, past 6 months or past month items for which questions varied from using free text responses (eg, participants reported the use of an amphetamine type stimulant in an ‘other substance’ field) to specifying explicit drug terminology (eg, ‘Amphetamines (speed, uppers, fast)’ or ‘Ice, methamphetamines’). We derived two binary variables to indicate whether participants had, in at least one wave, endorsed monthly or more frequent amphetamine use during adolescence (0 = no use/<monthly use, 1 = ≥monthly use) and during young adulthood (0 = no use/<monthly use, 1 = ≥monthly use).

Adolescent mental health difficulties were measured in each study as follows: the CHDS included the Composite International Diagnostic Interview [22] (CIDI) assessment of depression and anxiety; the ATP included the Short Mood and Feelings Questionnaire [23, 24, 25] (SMFQ); the VAHCS included assessment of common mental disorders with the Revised Clinical Interview Schedule [26] (CIS‐R); the IYDS included the SMFQ [23, 24, 25]. Young adult mental health difficulties were measured in each study as follows: the CHDS included the CIDI assessment of depression and anxiety; the ATP included the 21‐item Depression, Anxiety and Stress Scale [27] (DASS21); the VAHCS included the CIS‐R, symptoms of depression and anxiety using the 12‐item General Health Questionnaire [28, 29] (GHQ‐12) and the CIDI (CIDI‐auto [30] for major depressive disorder and CIDI‐short form [31] for anxiety disorder); the IYDS included the Kessler‐10 [32, 33] (K10). Based on validated cut‐points indicating elevated difficulties, we derived two binary variables indicating whether participants had reported mental health difficulties during adolescence (0 = no difficulties, 1 = any difficulties) and during young adulthood (0 = no difficulties, 1 = any difficulties).

#### Potential confounding factors

Potential confounding factors (Material S2, Table S2.2) were selected in line with the disjunctive cause criterion, based on a proposed causal relationship with either the exposure or outcome (or both) [34]. Indicators across a broad array of domains were selected from baseline or pre‐exposure time points, where possible, to ensure temporal precedence (ie, are not potential mediators in the causal pathway). The potential confounding factors and proposed causal pathways are visualised in directed acyclic graphs [35] (DAGs) for each research question (Material S2, Figure S2.1). Briefly, these included characteristics and behaviours at the participant level (ie, sex, academic performance, behaviour problems, antisocial behaviours, alcohol use, tobacco use and other illicit substance use), parent level (ie, alcohol use/attitudes, tobacco use/attitudes, country of birth/ethnicity, education level, separation/divorce and parent–child relationship quality) and peer level (ie, deviant peer affiliations). Further, to reduce the potential for reverse causation [34] we adjusted for adolescent levels of the young adult outcome. Continuous potential confounding factors were standardized (z‐scores) to enable harmonisation.

#### Statistical analyses

The analysis was not pre‐registered, and the results should be considered exploratory. All analyses were conducted in Stata 18 [36]. All models were conducted in the full sample and then stratified by male and female participants. First, the proportion of participants endorsing amphetamine use and mental health difficulties within and across adolescence and young adulthood were estimated. Second, we estimate associations between amphetamine use and mental health difficulties (and vice versa) as risk ratios (RRs) using G‐computation [37] procedures and Poisson regressions with robust error variance [38]. The G‐computation procedure involves fitting a regression model (using the *glm* command), and then using the model estimates to predict and then compare outcome values across exposure levels (using the *mimrgns* command) [39]. Models were run: (1) including study cohort (1 = CHDS, 2 = ATP, 3 = VAHCS, 4 = IYDS) as a categorical covariate [40]; (2) additionally accounting for all confounders except adolescent levels of the outcome; and (3) further accounting for adolescent levels of the outcome. Further, the G‐computation approach enabled the inclusion of interactions between exposure and both study cohort and sex in models. We further estimated the marginal probability of subsequent amphetamine use and mental health difficulties. The alignment between our analyses and a hypothetical ideal randomised control trial are presented in the Target Trial Emulation [41] tables (Material S3, Table S3.1 and Table S3.2).

Multiple imputation was used to handle missing data in the inferential analyses. Separate imputations were conducted within each cohort and for both males and females. Fifty complete datasets were imputed, based on a multivariate normal model [42]. Binary variables were imputed as continuous variables and then back transformed with adaptive rounding following imputation [43]. This approach has been shown to perform similarly to imputation models using chained equations [42]. Estimates were obtained by pooling results across the 50 imputed datasets using Rubin's rules [44].

We conducted several sensitivity analyses to examine the robustness of the conclusions drawn from our main analyses, whereby we repeated analyses: (1) in each cohort separately; (2) removing participants from the amphetamine variable base category who reported any amphetamine use to derive a non‐amphetamine‐using base category (*n* = 6750, 774 removed); and (3) using non‐imputed data.

## RESULTS

The analytic sample size was 7527 (3848 female), for which individual study contributions were: CHDS *n* = 1056 (534 female), ATP *n* = 1644 (820 female), VAHCS *n* = 1943 (1000 female) and IYDS *n* = 2884 (1489 female). In the CHDS, 71% of participants had two parents with less than tertiary education, 27% had parents who were separated and 11% reported Māori ethnicity. In the ATP, 27% of participants had two parents with a high school or lower education, 16% had parents who had separated and 28% had at least one non‐Australian born parent. In the VAHCS, 56% of participants had two parents with a high school or lower education, 20% had parents who had separated and 47% had at least one non‐Australian born parent. In the IYDS, 43% of participants had two parents with a high school or lower education, 16% had parents who had separated and 24% had a non‐Australian born respondent parent. Across all variables missing data ranged from between 0% to 9% (CHDS: 0%–1%, ATP: 0%–8%, VAHCS: 0%–9%, IYDS: 0%–7%).

### Amphetamine use and mental health difficulties

Table 2 shows the rates of amphetamine use (≥monthly) and mental health difficulties (any) within and across adolescence and young adulthood.

**TABLE 2:** Proportion of adolescent and young adult frequent amphetamine use and mental health difficulties within and across developmental periods.

	Total		Male		Female	
	*n*	% (95% CI)	*n*	% (95% CI)	*n*	% (95% CI)
≥Monthly amphetamine use						
Adolescent	149	1.97% (1.65, 2.30)	70	1.89% (1.41, 2.36)	79	2.06% (1.59, 2.52)
Young adult	757	10.06% (9.29, 10.84)	464	12.59% (11.32, 13.86)	293	7.64% (6.73, 8.55)
Any mental health difficulties						
Adolescent	3055	40.60% (39.48, 41.73)	1089	29.58% (28.08, 31.07)	1966	51.17% (49.57, 52.77)
Young adult	3557	47.27% (46.10, 48.45)	1477	40.11% (38.41, 41.82)	2080	54.13% (52.51, 55.76)
Adolescent amphetamine use and young adult mental health difficulties						
<Monthly adolescent amphetamine use						
No young adult mental health difficulties	3917	53.12% (51.92, 54.31)	2177	60.27% (58.54, 61.99)	1740	46.25% (44.60, 47.90)
Any young adult mental health difficulties	3458	46.88% (45.69, 48.08)	1436	39.73% (38.01, 41.46)	2023	53.75% (52.10, 55.40)
≥Monthly adolescent amphetamine use						
No young adult mental health difficulties	50	33.51% (25.09, 41.92)	28	40.06% (26.65, 53.47)	22	27.70% (17.14, 38.27)
Any young adult mental health difficulties	99	66.49% (58.08, 74.91)	42	59.94% (46.53, 73.35)	57	72.30% (61.73, 82.86)
Adolescent mental health difficulties and young adult amphetamine use						
No adolescent mental health difficulties						
<Monthly young adult amphetamine use	4073	91.14% (90.16, 92.12)	2298	88.62% (87.14, 90.10)	1775	94.63% (93.53, 95.73)
≥Monthly young adult amphetamine use	396	8.86% (7.88, 9.84)	295	11.38% (9.90, 12.86)	101	5.37% (4.27, 6.47)
Any adolescent mental health difficulties						
<Monthly young adult amphetamine use	2694	88.18% (86.88, 89.47)	921	84.53% (81.97, 87.09)	1773	90.20% (88.79, 91.60)
≥Monthly young adult amphetamine use	361	11.82% (10.53, 13.12)	168	15.47% (12.91, 18.03)	193	9.80% (8.40, 11.21)

The number of participants reporting ≥monthly amphetamine use was 149 (1.97%, 95% CI = 1.65, 2.30) in adolescence and 757 (10.06%, 95% CI = 9.29, 10.84) in young adulthood. The proportion of male and female participants reporting ≥monthly amphetamine use was similar during adolescence (male: 1.89%, 95% CI = 1.41, 2.36; female: 2.06%, 95% CI = 1.59, 2.52), but in young adulthood was somewhat elevated in males (12.59%, 95% CI = 11.32, 13.86) compared with females (7.64%, 95% CI = 6.73, 8.55).

A total of 3055 participants reported mental health difficulties in adolescence (40.60%, 95% CI = 39.48, 41.73) and 3557 in young adulthood (47.27%, 95% CI = 46.10, 48.45). Mental health difficulties were reported by a higher proportion of females than males in both adolescence (males: 29.58%, 95% CI = 28.08, 31.07; females: 51.17%, 95% CI = 49.57, 52.77) and young adulthood (males: 40.11%, 95% CI = 38.41, 41.82; females: 54.13%, 95% CI = 52.51, 55.76).

Mental health difficulties were experienced by 66.49% (95% CI = 58.08, 74.91) of young adults who reported ≥monthly amphetamine use in adolescence, compared with 46.88% (95% CI = 45.69, 48.08) among young adults who did not. In males, the proportion of mental health difficulties in young adulthood was about one‐half higher in those who reported ≥monthly amphetamine use in adolescence compared with those who did not (<monthly use 39.73% vs. ≥ monthly 59.94%). In females, this was slightly lower, with mental health difficulties in young adulthood about one‐third higher in those reporting adolescent ≥monthly amphetamine use compared with those who did not (< monthly use 53.75 vs. ≥ monthly use 72.30%).

Monthly or more frequent amphetamine use was reported by 11.82% (95% CI = 10.53, 13.12) of young adults who experienced adolescent mental health difficulties, compared with 8.86% (95% CI = 7.88, 9.84) who did not. In males, the proportion of ≥monthly amphetamine use in young adulthood was around one‐third higher in those who reported adolescent mental health difficulties (no difficulties 11.38% vs. any difficulties 15.47%). In females, however, differences were much more substantial, with ≥monthly amphetamine use in those who reported adolescent mental health difficulties being close to double that of those who did not report mental health difficulties in adolescence (no difficulties 5.37% vs. any difficulties 9.80%).

### Associations between amphetamine use and mental health difficulties

**FIGURE 1:**
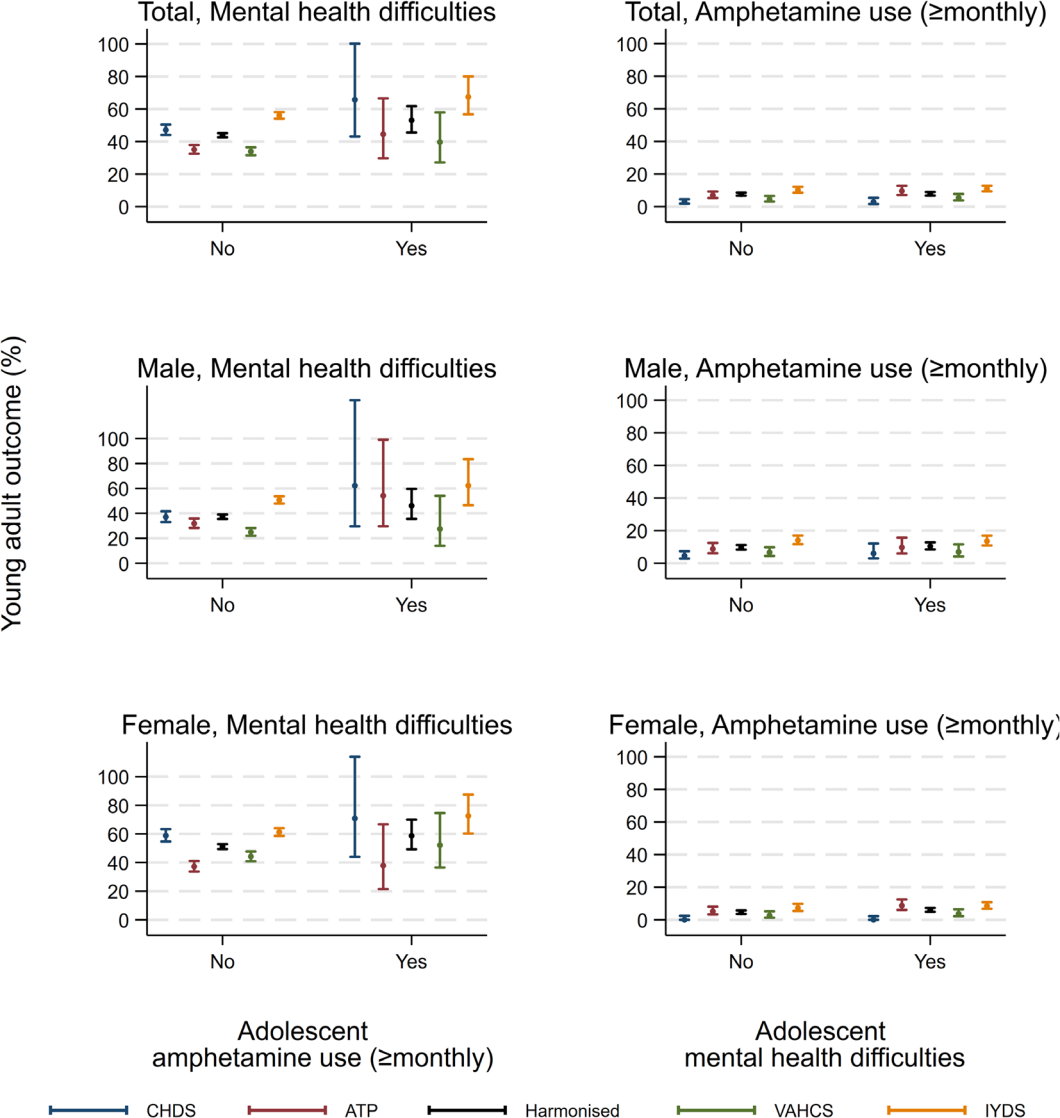
Estimated young adult mental health difficulties and ≥monthly amphetamine use by adolescent exposure status after accounting for all potential confounding factors and interactions between exposure, cohort and participant sex (when present in the model). ATP, Australian temperament project; CHDS, Christchurch health and development study; IYDS, International Youth and Development Study; VAHCS, Victorian adolescent health cohort study.

Table 3 presents RRs for the association between ≥monthly amphetamine use and mental health difficulties across adolescence and young adulthood. Further, Figure 1 presents the estimated proportion of young adult mental health difficulties and ≥monthly amphetamine use across adolescent exposure status in the fully adjusted models.

**TABLE 3:** Associations between frequent amphetamine use and mental health difficulties across subsequent developmental periods in the harmonised data.

	Cohort adjustment^a^		Potential confounding factor adjustment^b^		Adolescent outcome adjustment^c^	
	RR	95% CI	RR	95% CI	RR	95% CI
	Total (*n* = 7527)					
Outcome: young adult mental health difficulties						
Adolescent amphetamine use						
<Monthly	(base)		(base)		(base)	
≥Monthly	1.42	(1.23, 1.63)	1.25	(1.07, 1.47)	1.21	(1.04, 1.41)
Outcome: young adult ≥monthly amphetamine use						
Adolescent mental health difficulties						
None	(base)		(base)		(base)	
Any	1.25	(1.05, 1.49)	1.21	(1.00, 1.47)	1.19	(0.99, 1.45)

	Male (*n* = 3684)					
Outcome: young adult mental health difficulties						
Adolescent amphetamine use						
<Monthly	(base)		(base)		(base)	
≥Monthly	1.54	(1.21, 1.97)	1.26	(0.96, 1.67)	1.24	(0.95, 1.60)
Outcome: young adult ≥monthly amphetamine use						
Adolescent mental health difficulties						
None	(base)		(base)		(base)	
Any	1.29	(1.02, 1.64)	1.08	(0.85, 1.38)	1.08	(0.85, 1.37)

	Female (*n* = 3843)					
Outcome: young adult mental health difficulties						
Adolescent amphetamine use						
<Monthly	(base)		(base)		(base)	
≥Monthly	1.32	(1.11, 1.57)	1.20	(1.00, 1.44)	1.15	(0.96, 1.37)
Outcome: young adult ≥monthly amphetamine use						
Adolescent mental health difficulties						
None	(base)		(base)		(base)	
Any	1.69	(1.27, 2.24)	1.36	(1.02, 1.83)	1.33	(0.99, 1.78)

*Note*: Models include interactions between exposure, cohort and participant sex when variables are present in the model.

Abbreviation: RR, risk ratio.

^a^ Includes adjustment for cohort.

^b^ Includes adjustment for cohort and potential confounding factors excluding adolescent outcome.

^c^ Includes adjustment for cohort and potential confounding factors including adolescent outcome.

In the fully adjusted model, evidence suggested that the risk of mental health difficulties in young adulthood was 21% greater (RR = 1.21, 95% CI = 1.04, 1.41) in those who reported ≥monthly amphetamine use in adolescence compared with those who did not. RRs were slightly stronger when models were estimated without potential confounding factors (RR = 1.42, 95% CI = 1.23, 1.63) or without adjustment for adolescent mental health difficulties (RR = 1.25, 95% CI = 1.07, 1.47).

Further, in the fully adjusted model, evidence suggested that the risk of ≥monthly amphetamine use in young adulthood was 19% greater (RR = 1.19, 95% CI = 0.99, 1.45) in those who reported mental health difficulties in adolescence compared with those who did not, although it is noted that the CI marginally overlapped the null value. RRs were slightly stronger when models were estimated without confounders (RR = 1.25, 95% CI = 1.05, 1.49) or without adjustment for adolescent ≥monthly amphetamine use (RR = 1.21, 95% CI = 1.00, 1.47).

When associations were examined in male and female participants separately, effect estimate CIs overlapped with both the null value and the CI of other estimates, limiting the evidence for associations and differences among associations. Nevertheless, the estimated risk of mental health difficulties in young adulthood in those who reported ≥monthly amphetamine use in adolescence, compared with those who did not, was slightly stronger in males (RR = 1.24, 95% CI = 0.95, 1.60) than females (RR = 1.15, 95% CI = 0.96, 1.37). Comparatively, the estimated risk of ≥monthly amphetamine use in young adulthood in those who reported mental health difficulties in adolescence compared with those who did not, was slightly more evident in females (RR = 1.33, 95% CI = 0.99, 1.78) than males (RR = 1.08, 95% CI = 0.85, 1.37).

### Sensitivity analyses

Sensitivity analyses broadly supported the conclusions drawn from our primary analyses (Material S4). A similar pattern of amphetamine use and mental health difficulties across adolescence and young adulthood was found within each individual cohort, although the proportion of ≥monthly amphetamine use in young adulthood was higher in the ATP and IYDS cohorts, which assessed past month use, than in the CHDS and VAHCS cohorts, which assessed use over a longer period (Table S4.1). Further, RRs were relatively similar across each cohort, although almost all CIs overlapped with the null value (Table S4.2). RRs were also similar when participants were removed from the amphetamine base category who reported any recent amphetamine use (Table S4.3). Additionally, when we repeated models using the non‐imputed data RRs were similar (Table S4.4).

## DISCUSSION

Using harmonised data from four mature Australasian cohort studies (the MISUse Consortium) we found evidence in support of a bidirectional association between amphetamine use (≥monthly) and mental health difficulties from adolescence to young adulthood. Specifically, amphetamine use in adolescence increased the risk of mental health difficulties in young adulthood by 21%, and mental health difficulties in adolescence increased the risk of subsequent amphetamine use in early adulthood by 19%, albeit with slightly less precision. Our findings support and extend prior meta‐analytic evidence which has suggested an association between (meth)amphetamine use and depression (k = 6, adjusted OR = 1.3) [6], with some differentiation of effects between cross‐sectional (k = 4; adjusted OR = 1.66) and longitudinal evidence (k = 2; adjusted OR = 1.18) [7]. Further, given the application of best practice casual modelling tools, the current findings may suggest a possible causal effect of both adolescent amphetamine use and mental health difficulties, although the inherent limitations in making such inferences from non‐causal designs should temper certainty in the conclusions.

Although evidence from our study suggests that there may be differences in the prominence of directional pathways (ie, for males the strongest association was from amphetamine use to mental health difficulties, whereas for females the strongest association was from mental health difficulties to amphetamine use), we note that these conclusions should remain tentative given the imprecision in our effect estimates. Nevertheless, current findings align with prior related diagnostic and help‐seeking evidence. For instance, in terms of comorbid substance use diagnoses, women, in comparison to men, often present with primary (as opposed to secondary) diagnoses of mental health problems. This may suggest that women with mental health problems are more likely than men to use substances to self‐medicate and are, therefore, at higher risk for also developing secondary substance use disorders [45]. Further, from a help‐seeking perspective, evidence has suggested that men are more likely to engage in substance use treatment services [46], whereas women are more likely to engage with mental health professionals [47]. Moreover, given the higher proportions of amphetamine use and mental health difficulties found in males and females, respectively, both in the current study and reported elsewhere [48, 49], future work should continue to examine the relative importance and impact of these pathways.

The evidence supporting a possible causal relationship between amphetamine use and mental health difficulties, and vice versa, has implications for both population and clinical strategies. The principle of proportionate universalism is that ‘actions should be universal, but with an intensity and a scale that is proportional to the level of disadvantage’ [50], which suggests that prevention strategies should be made universally available at a population level, but also enriched and more extensively resourced in groups of adolescents at elevated risk for substance use or mental health problems. Further, given that reducing both mental health and substance use in adolescence is likely to yield the largest improvements in early adult outcomes, the use of transdiagnostic models that target common risk factors to both conditions, such as social resilience, may be both efficient and effective [51, 52]. Our findings suggest that strategies to reduce the burden of disease from mental health problems should consider the role of amphetamines alongside other substances that are recognised as causing depression, notably alcohol [53].

Although our findings point to notable effect sizes at a population level, future research should seek to further delineate these relationships. For instance, prior meta‐analytic evidence has identified stronger risk relationships between methamphetamine use and depression when examining methamphetamine use disorder (Diagnostic and Statistical Manual of Mental Disorders classification), in contrast to any use [7]. Such differentiations may be also evident in those with clinically diagnosed mental health problems. This may also extend to other operationalisations of amphetamine use and mental health, which capture time‐related factors such as time since first and last exposure or the cumulative impact across multiple age periods. For example, several examinations in population‐based cohort studies have revealed the negative impact of persistent patterns of substance use and mental health problems on subsequent outcomes within and across generations [54, 55, 56, 57, 58, 59, 60]. Similarly, additional research is needed to explore the mechanisms by which amphetamine use causes mental health problems, and vice versa. For example, the effect of amphetamine use on later mental health difficulties may be driven by biological pathways involved in withdrawal [5], but may also reflect impacts on functioning more broadly (eg, effects on relationships, work/education) because of ongoing use [8].

Although the MISUse Consortium brings several strengths to the study of low prevalence illicit drug use in particular, mature cohort studies contain sources of selection, measurement and confounding bias that require consideration [61]. Although multiple imputation was used to minimise missing data bias, it is likely that there was some selective attrition of the most vulnerable individuals, such as those with high levels of substance use or mental health difficulties. Nevertheless, rates of amphetamine use and mental health problems were slightly higher than population‐level prevalence estimates [48, 62], a pattern that has been noted within prior cohort studies [63]. Further, given that data are derived from cohorts established between the 1970s and 2000s, albeit with birth years approximately within a decade of one another, contemporary data collections remain important for understanding the rapidly evolving substance use landscape.

Additionally, across the included cohort studies there are several differences in construct measurement, which may have resulted in some imprecision following data harmonisation, although the inclusion of a cohort identified in analyses helps to address across‐cohort measurement biases [40]. For instance, there was variation in the assessment of ‘monthly’ amphetamine use, for which measures differed in terms of capturing an average of monthly use versus capturing use from the past month. Similarly, the derivation of binary variables and collapsing several waves within each developmental period can create heterogeneity in exposure and outcome operationalisation, such that groups include variation in frequency/symptoms and combinations of persistent versus transient cases. Nevertheless, despite these (and other) measurement differences, supplementary analyses in each cohort generally supported the overall harmonised findings, although with notably less power and precision. It is noted that the assessment of amphetamine use (and our indicators of other substance use behaviours) did not capture the non‐prescribed use of prescription medications as data on such use was not consistently captured among the cohorts, albeit such use has become increasingly common among young Australians over the past decade [49]. Finally, although we implemented several best‐practice tools for making causal inferences in observational data, including the Target Trial Emulation, DAGs and G‐computation, causal inference in observational data requires strong assumptions, such as no residual confounding. Although we included a broad range of key potential confounding factors, unmeasured confounding remains an issue, requiring future work to replicate these findings.

In conclusion, amphetamine use and mental health difficulties appear to have a bidirectional association across adolescence and young adulthood, although estimates were slightly imprecise for the impact on young adult amphetamine use. The importance of these pathways may vary by sex, although the imprecision in estimates within male and female participants does warrant some caution in the interpretation. Consistent with the methodological approaches used, our findings may suggest a possible causal effect, however, given the assumptions inherent to causal modelling efforts in observational data, further replication is warranted. We suggest that a comprehensive approach to breaking this interactive pattern of maladaptive development would involve coordinated public health and health services responses that placed great priority on assessment and intervention in adolescent populations. Nevertheless, current findings suggest the intervention efforts targeting adolescent amphetamine use and mental health difficulties may see improvements in young adulthood outcomes.

## AUTHOR CONTRIBUTIONS

**Christopher J. Greenwood:** Conceptualization (lead); data curation (lead); formal analysis (lead); visualization (lead); writing—original draft (lead); writing—review and editing (lead). **James Foulds:** Conceptualization (equal); funding acquisition (equal); investigation (equal); project administration (equal); writing—original draft (equal); writing—review and editing (equal). **Rebecca McKetin:** Conceptualization (equal); writing—original draft (equal); writing—review and editing (equal). **Stephanie R. Aarsman:** Data curation (equal); formal analysis (equal); writing—review and editing (equal). **Delyse Hutchinson:** Conceptualization (equal); investigation (equal); writing—review and editing (equal). **Jessica Kerr:** Conceptualization (equal); project administration (equal); writing—review and editing (equal). **Jessica A. Heerde:** Conceptualization (equal); investigation (equal); writing—review and editing (equal). **John W. Toumbourou:** Conceptualization (equal); funding acquisition (equal); investigation (equal); project administration (equal); writing—review and editing (equal). **Joseph M. Boden:** Conceptualization (equal); data curation (equal); funding acquisition (equal); investigation (equal); project administration (equal); writing—review and editing (equal). **Tim Slade:** Conceptualization (equal); writing—review and editing (equal). **Yvonne Bonomo:** Conceptualization (equal); writing—review and editing (equal). **Primrose Letcher:** Conceptualization (equal); funding acquisition (equal); investigation (equal); project administration (equal); writing—review and editing (equal). **Craig A. Olsson:** Conceptualization (equal); funding acquisition (equal); investigation (equal); project administration (equal); supervision (lead); writing—original draft (equal); writing—review and editing (equal).

## FUNDING INFORMATION

The Christchurch Health and Development Study was funded by the Health Research Council of New Zealand (Programme grant 16/600). The Australian Temperament Project was supported primarily through Australian grants from the Melbourne Royal Children's Hospital Research Foundation, National Health and Medical Research Council (NHMRC), Australian Research Council (ARC) and the Australian Institute of Family Studies. Funding for this work was supported by grants from the Australian Research Council (DP130101459; DP160103160; DP180102447) and the NHMRC of Australia (APP1082406). The Victorian Adolescent Health Cohort Study was supported by NHMRC (APP1063091; APP1008273; APP1157378), Australian Rotary Health, Colonial Foundation, Perpetual Trustees, Financial Markets Foundation for Children (Australia), Royal Children's Hospital Foundation and the Murdoch Children's Research Institute The International Youth Development Study was supported in part by grants from the National Institute on Drug Abuse (R01DA012140), the National Institute on Alcoholism and Alcohol Abuse (R01AA017188), the NHMRC (491241) and the ARC (DP109574, DPO663371 and DPO877359). C.G. receives salary and research support from an Alfred Deakin Research Fellowship at Deakin University. J.H. receives salary and research support from a NHMRC Investigator Grant (GNT2007722). She holds a Dame Kate Campbell Fellowship awarded by the Faculty of Medicine, Dentistry and Health Sciences at The University of Melbourne. C.A.O. and D.M.H. were supported by NHMRC fellowships (Investigator grant APP1175086, APP1197488).

## DECLARATION OF INTERESTS

None.

## Supporting information


**Material S1.** Description of cohort studies involved.
**Material S2.** Description of amphetamine use, common mental health problem, and potential confounding factor measures.
**Material S3.** Target Trial Emulation templates.
**Material S4.** Sensitivity analyses.

## Data Availability

Ethics approvals for the CHDS, ATP, VAHCS, and IYDS do not permit the data to be made publicly available due to limitations of participant consent and concerns regarding potential re‐identifiability. For the CHDS, data availability inquiries can be directed to the Principal Scientist of the CHDS: Prof. Joseph M. Boden (joseph.boden@otago.ac.nz). For the ATP, VAHCS, and IYDS, the dataset subset can be made available to a named individual for the purpose of replication of research findings. Requests to access the dataset can be submitted through our institutional data access protocol: https://lifecourse.melbournechildrens.com/data-access/.
