# Supplementary material for: Amphetamine use and mental health difficulties across adolescence and young adulthood: An integrative data analysis of four Australasian cohort studies
Source: Addiction. 2025 Mar 15;120(8):1623–33. doi: 10.1111/add.70033 (PMC12215235; doi:10.1111/add.70033)
Supplement: Supplementary file 1 — Material S1. Description of cohort studies involved. Material S2. Description of amphetamine use, common mental health problem, and potential confounding factor measures. Material S3. Target Trial Emulation templates. Material S4. Sensitivity analyses. [file ADD-120-1623-s001.docx]

**Amphetamine use and mental health difficulties across adolescence and young adulthood: An integrative data analysis of four Australasian cohort studies.**

# Supplementary materials

Supplementary material 1: Description of cohort studies involved.

Additional information about the four longitudinal cohorts involved in this study is provided below.

***Christchurch Health and Development Study (CHDS).*** The CHDS is a birth cohort of 1265 children born in the Christchurch (New Zealand) urban region in 1977. These children included 97% of all live births occurring during the recruitment period (April-August 1977). The cohort has now been studied on a total of 24 occasions (birth, 4 months, yearly between 1-16 years, 18 years, 21 years, 25 years, 30 years, 35 years, 40 years). Data on a broad range of physical health, emotional and behavioural outcomes have been gathered from a range of sources. Primary adolescent variables are drawn from the age 17–18-year assessment and young adult variables are drawn from the age 20-21-, 24-25-, and 29–30-year assessments.

***Australian Temperament Project (ATP).*** The ATP is a cohort of 2443 infants (aged 4-8 months) born in Victoria (Australia) recruited in 1983. 67 Local Government Areas (LGAs) in the state of Victoria were randomly selected based on Australian Bureau of Statistics, to provide a representative community sample. Parents of every 4–8-month-old infant who visited one of the selected centres between 22nd April and 6th May (1983) were invited to participate. Families completed mail surveys in 1983, 1984, and 1985 and then approximately every 2 years until children were 19-20 years of age (13 waves, 1983-2002), and every 4 years thereafter (3 waves, 2006-2014; from 2010, participants could also opt to complete the survey online). Primary adolescent variables are drawn from the age 13-14-, 15-16-, and 17–18-year assessment and young adult variables are drawn from the age 19-20-, 23-24-, and 28–29-year assessments.

***Victorian Adolescent Health Cohort Study (VAHCS).*** The VAHCS is a longitudinal study of a representative sample of mid-secondary adolescent recruited in 1992 from Victoria (Australia). Participants were recruited via schools at the end of Year 9 (wave 1, mean age 14.9 years) or the start of Year 10 (wave 2, mean age 15.5 years), and were assessed on four further occasions during adolescence: wave 3 (mean age 15.9 years) - wave 6 (mean age 17.4 years); with a further five follow-ups in adulthood: wave 7 (mean age 20.7 years) - wave 11 (mean age 42.6 years). Primary adolescent variables are drawn from the age 15.5-, 15.9-, 16.4-, 16.9-, and 17.4-year assessment and young adult variables are drawn from the age 20.7-, 24.1-, and 29.1-year assessments.

***International Youth Development Study – Victorian Arm (IYDS).*** The IYDS is a state representative cohort study of a students in Grade 5, 7, and 9 (approx. age 11-, 13-, and 15-years) recruited in 2002 from Victoria (Australia). A two-stage cluster sampling approach for school recruitment was used, whereby schools were randomly selected in the first stage, and a target classroom within each school was randomly selected in the second stage. Within each state and grade level, public and private schools containing Grades 5, 7, or 9 were randomly selected using a probability proportionate to grade-level size-sampling procedure. 165 classes in 152 schools agreed to participate in the study. Participants completed a total of 10 surveys approximately every year from 2002 to 2008 (no survey in 2005), then every two years from 2010 to 2014, and with a final collection in 2018. Primary adolescent variables are drawn from the waves 1 to 6 (spanning age 11- to 17-years) assessments and young adult variables are drawn from the waves 7 to 10 (spanning age 19- to 29- years) assessments.

Supplementary material 2. Description of amphetamine use, common mental health problem, and potential confounding factor measures.

A summary of the amphetamine use and common mental health problem measures is presented in Table 2.1.

***Amphetamine use.***

The CHDS (1 wave adolescence [age 17-18 years], 3 waves young adulthood [age 21-30 years]) assessed the number of times used in the past year. Response options varied between a continuous response (age 17-18- [including age 16-17 years] and 20-21-years) and a categorical response (never used, once or twice only, <monthly, at least monthly, at least weekly, several times a week, daily, several times a day; age 24-25- and 29-30-years). Monthly use was derived as reporting amphetamine use ≥12 times or ≥‘at least monthly’ at any wave within each developmental period.

The ATP (2 waves adolescence [age 15-18 years], 3 waves young adulthood [age 19-28 years]) assessed the number of days used in the past month. Monthly use was derived as reporting amphetamine use ≥1 day in the past month at any wave within each developmental period.

The VAHCS (5 waves adolescence [age 15-17 years], 3 waves young adulthood [age 20-29 years]) primarily assessed the frequency of use in the past year or 6 months. Response options varied between adolescent (never, not in the past 6 months, a few times a year, monthly, weekly, daily) and young adult (less than once a month, 1 to 3 days a month, 1 or 2 days a week, 3 or 4 days a week, almost every day) assessments. Monthly use was derived as reporting amphetamine use ≥‘monthly’ or ≥‘1 to 3 days a month’ at any wave within each developmental period.

The IYDS (6 waves adolescence [age 13-17 years], 4 waves young adulthood [age 19-29 years]) assessed the number of times used in the past month. Response options were never, 1 or 2 times, 3 to 5 times, 6 to 9 times, 10 to 19 times, 20-29 times, 30-39 times, 40 or more times. Monthly use was derived as reporting amphetamine use ≥‘1 or 2 times’ at any wave within each developmental period.

***Mental health difficulties.***

The CHDS (1 wave adolescence [age 17-18 years], 3 waves young adulthood [age 21-30 years]) included the Composite International Diagnostic Interview (1) (CIDI) assessment of depression and anxiety.

The ATP (3 waves adolescence [age 13-18 years], 3 waves young adulthood [age 19-28 years]) included during adolescence the Short Mood and Feelings Questionnaire (2,3) (SMFQ; elevated symptoms ≥11 (4)) which was categorised to indicate elevated symptom levels) and during young adulthood the 21-item Depression, Anxiety, and Stress Scale (5) (DASS21; mild to extreme symptoms depression ≥5, anxiety≥4, stress ≥8).

The VAHCS (5 waves adolescence [age 15-17 years], 3 waves young adulthood [age 20-29 years]) included during adolescence assessment of common mental disorders with the Revised Clinical Interview Schedule (6) (CIS-R; presence of common mental disorder ≥12) and during young adulthood the CIS-R at age 20, symptoms of depression and anxiety using the 12-item General Health Questionnaire (7,8) (GHQ-12; high symptoms ≥3) at ages 24 and 29 years, and the Composite International Diagnostic Interview (CIDI; CIDI-auto (9) for major depressive disorder and CIDI-short form (10) for anxiety disorder) additionally at age 29 years.

The IYDS (6 waves adolescence [age 13-17 years], 4 waves young adulthood [age 19-29 years]) included in adolescence the SMFQ (2,3) (elevated symptom levels ≥11 (4)) and in young adulthood the Kessler-10 (11) (K10; mild to severe mental disorder ≥20 (12)) assessment of psychological distress.

| Table 2.1. Amphetamine and common mental health measurement | | | | | | | | | | | |
| --- | --- | --- | --- | --- | --- | --- | --- | --- | --- | --- | --- |
| CHDS | | | ATP | | | VAHCS | | | IYDS | | |
| Age | Measurement | | Age | Measurement | | Age | Measurement | | Age | Measurement | |
|  | Amphetamine Use | Common mental  health problems |  | Amphetamine Use | Common mental  health problems |  | Amphetamine Use | Common mental  health problems |  | Amphetamine Use | Common mental  health problems |
| Adolescence | | | | | | | | | | | |
| 17-18 | Past year frequency: number of times (includes assessment of 16-17 years) | CIDI | 13-14 | No assessment | SMFQ | 15.5 | How often: never, not in the past 6 months, a few times a year, monthly, weekly, daily | CIS-R | 13m, 15o | Past month: never, 1 or 2 times, 3 to 5 times, 6 to 9 times, 10 to 19 times, 20-29 times, 30-39 times, 40 or more times | SMFQ |
|  |  |  | 15-16 | No assessment | SMFQ | 15.9 | As above | CIS-R | 14m, 16o | Past month: never, 1 or 2 times, 3 to 5 times, 6 to 9 times, 10 to 19 times, 20-29 times, 30-39 times, 40 or more times | SMFQ |
|  |  |  | 17-18 | Past month frequency: number of days | SMFQ | 16.4 | As above | CIS-R | 13y, 15m | As above | SMFQ |
|  |  |  |  |  |  | 16.9 | As above | CIS-R | 15y | As above | SMFQ |
|  |  |  |  |  |  | 17.4 | As above | CIS-R | 16y | As above | SMFQ |
|  |  |  |  |  |  |  |  |  | 17y | As above | SMFQ |
| Young adulthood | | | | | | | | | | | |
| 20-21 | Past year frequency: number of times | CIDI | 19-20 | Past month frequency: number of days | DASS21 | 20.7 | No assessment | CIS-R | 19y, 21m, 23o | Past month: never, 1 or 2 times, 3 to 5 times, 6 to 9 times, 10 to 19 times, 20-29 times, 30-39 times, 40 or more times | K10 |
| 24-25 | Past year frequency: never used, once or twice only, <monthly, at least monthly, at least weekly, several times a week, daily, several times a day | CIDI | 23-24 | As above | DASS21 | 24.1 | Past year frequency: less than once a month, 1 to 3 days a month, 1 or 2 days a week, 3 or 4 days a week, almost ever day | GHQ-12 | 21y, 23m, 25o | As above | K10 |
| 29-30 | As above | CIDI | 27-28 | As above | DASS21 | 29.1 | As above | GHQ-12, CIDI | 23y, 25m, 27o | As above | K10 |
|  |  |  |  |  |  |  |  |  | 29m | As above | K10 |
| Notes: CHDS=Christchurch Health and Development Study; ATP=Australian Temperament Project; VAHCS=Victorian Adolescent Health Cohort Study; IYDS=International Youth and Development Study; IYDS age: y=youngest cohort, m=middle cohort, o=oldest cohort | | | | | | | | | | | |

***Potential confounding factors.*** A summary of potential confounding factors is provided in Table 2.2. The proposed Directed Acyclic Graph corresponding to the assignment emulation is presented in Figure 2.1.

| Table 2.2. Description of potential confounding factors | | | | |
| --- | --- | --- | --- | --- |
|  | CHDS | ATP | VAHCS | IYDS |
| Participant Level |  |  |  |  |
| Sex | male, female | male, female | male, female | male, female |
| Academic performance | Grade point average; teacher-report; age 11-13 years. | Math and reading skills; teacher-report; age 7-8 and 11-12 years. (13,14) | Compared with other students at your year level how well do you do at school?; child-report; age 15.5 years. | Grades better than most students; child-report; age 11-15 years. |
| Behaviour problems | Conduct/attention problems; parent-report; age 7-9 years. (15) | Anxious/fearful and hyperactivity; parent-report; age 5-6 to 12-13 years. (16) | Impulsivity; child-report; age 15.5 years. (17) | Attention and impulsivity; child-report; age 11-15 years. (18) |
| Antisocial behaviour | Count of number of self-reported crimes; child-report; age 18 years. (19) | Frequency of antisocial behaviours; child-report; age 13-14 years. (20) | Count of number of antisocial behaviours; child-report; age 15.5 years. (20) | Frequency of antisocial behaviours; child-report; child-report; age 11-15 years. (21) |
| Alcohol use | Any alcohol consumption (past 12 months); child-report; age 15 years. | Ever used alcohol; child-report; age 13-14 years. | Current alcohol use; child-report; age 15.5 years. | Ever used alcohol; child-report; age 11-15 years. |
| Tobacco use | Any tobacco use; child-report; age 15 years. | Ever used tobacco; child-report; age 13-14 years. | Current tobacco use; child-report; age 15.5 years. | Ever used tobacco; child-report; age 11-15 years. |
| Other illicit substance use | Any adolescent cannabis, cocaine, ecstasy, hallucinogen, or opioid use; self-report; age 15-18 years. | Any adolescent cannabis, cocaine, ecstasy, hallucinogen, or opioid use; self-report; age 13-14 to 17-18 years. | Any adolescent cannabis, cocaine, ecstasy, hallucinogen, or opioid use; self-report; age 15.5-17.4 years. | Any adolescent cannabis, cocaine, ecstasy, hallucinogen, or opioid use; self-report; age 11-17 years. |
| Parent Level |  |  |  |  |
| Alcohol use | Parental history of alcohol problems; parent-report; age 11 years. | Frequency of parent alcohol use; parent-report; age 13-14. | Frequency of parent alcohol use; child-report; age 14.9-17.4 years. | Parent attitudes towards alcohol; child-report; age 11-15 years. |
| Tobacco use | Mother/Father tobacco smoking status; parent-report; age 10 years. | Frequency of parent tobacco use; parent-report; age 13-14 years | Frequency of parent tobacco use; child-report; age 14.9-17.4 years. | Parent attitudes towards tobacco; child-report; age 11-15 years. |
| Country of birth/ethnicity | Not Māori ethnicity, Māori ethnicity; parent-report; birth. | Both parents born in Australia, At least one parent not born in Australia; parent-report. | Both parents born in Australia, At least one parent not born in Australia; parent-report; age 15.5-24.1 years. | Respondent parent born in Australia, Respondent parent not born in Australia; parent-report. |
| Education level | At least one parent with high education (≥tertiary), Both parents with low (<tertiary) education; parent-report; birth. | At least one parent with high education (>year 12), Both parents with low (≤year 12) education; parent-report; age 4-8 months to 12-13 years. | At least one parent with high education (>year 12), Both parents with low (≤year 12) education; child-report; age 15.5-17.4 years. | At least one parent with high education (>year 12), Both parents with low (≤year 12) education; parent-report; age 11-15 years. |
| Separation | Parent experience of separation, parent-report; age 1-10 years. | Parent experience of divorce/separation; parent-report; age 4-8 months to 12-13 years. | Parent experience of divorce/separation; child-report; age 15.5-17.4 years. | Parent experience of divorce/separation; parent-report; age 11-15 years. |
| Parent-child relationship quality | Parental attachment; child-report; age 14 years. (22) | Parent-child attachment quality; child-report; age 13-14 years. (22) | Perceived stress with family and living arrangements; child-report, age 15.5 years. (23) | Parent-child attachment quality; child-report; age 11-15 years. (24,25) |
| Peer Level |  |  |  |  |
| Deviant peer affiliation | Number of friends who use drugs and break the law; child-report; age 15 years. | Frequency of affiliation with deviant peers; child-report; age 13-14. | Number of friends who use alcohol, tobacco and drugs; child-report; age 15.5 years. | Number of peers engaged in antisocial behaviour; child-report; age 11-15 years. |
| Notes: CHDS=Christchurch Health and Development Study; ATP=Australian Temperament Project; VAHCS=Victorian Adolescent Health Cohort Study; IYDS=International Youth and Development Study. | | | | |

Figure 2.1. Directed Acyclic Graphs for research questions examining a) the causal effect of adolescent ≥monthly amphetamine use on subsequent young adulthood mental health difficulties, and b) the causal effect of adolescent mental health difficulties on subsequent young adulthood ≥monthly amphetamine use.

Supplementary material 3. Target Trial Emulation templates

We completed Target Trial Emulated templates for our research questions examining 1) a causal relationship between adolescent amphetamine use and young adult mental health difficulties (table 3.1); 2) a causal relationship between adolescent mental health difficulties and young adult amphetamine use (table 3.2).

| Table 3.1. Target Trial Emulation: What is the causal effect of adolescent ≥monthly amphetamine use on subsequent young adulthood mental health difficulties | | | | | |
| --- | --- | --- | --- | --- | --- |
| Protocol component | Target trial specification | Emulation CHDS | Emulation ATP | Emulation VAHCS | Emulation IYDS |
| Eligibility criteria | Adolescent (age 10-18 years) males and females from Australia and New Zealand across the late ‘70s into the early ‘90s (1977 to 1992). | Adolescent (age 17-18 years) males and females born in Christchurch, New Zealand in 1977. | Adolescent (age 17-18 years) males and females born in Victoria, Australia in 1983. | Adolescent (age 15-17.4 years) males and females living in Victoria, Australia as adolescents (born on average in 1978). | Adolescent (age 11-17 years) males and females living in Victoria, Australia as adolescents (born in 1988-1992). |
|  |  |  |  |  |  |
| Sub-group analyses | Male and female samples. | Male and female samples. | Male and female samples. | Male and female samples. | Male and female samples. |
|  |  |  |  |  |  |
| Treatment strategies ^*^ | Intervention arm: participants use amphetamines ‘monthly’ at least once across adolescence.  Comparator arm: no intervention (no ‘monthly’ amphetamine use). | Intervention: any ‘monthly’ (≥12 times in past year) amphetamine use in adolescence (age 17-18 years).  Comparator: No ‘monthly’ amphetamine use across adolescence. | Intervention: any ‘monthly’ (≥1 day in past month) amphetamine use in adolescence (age 17-18 years).  Comparator: No ‘monthly’ amphetamine use across adolescence. | Intervention: any ‘monthly’ (≥monthly in past 6 months) amphetamine use in adolescence (age 15.5-17.4 years).  Comparator: No ‘monthly’ amphetamine use across adolescence. | Intervention: any ‘monthly’ (≥1 or 2 times in past month) amphetamine use in adolescence (age 11-17 years).  Comparator: No ‘monthly’ amphetamine use across adolescence. |
|  |  |  |  |  |  |
| Assignment procedures | Participants will be randomly assigned to either treatment strategy and will be aware of the strategy to which they have been assigned. | Selection of confounders.  Approach to confounder adjustment: G-computation. | Selection of confounders.  Approach to confounder adjustment: G-computation. | Selection of confounders.  Approach to confounder adjustment: G-computation. | Selection of confounders.  Approach to confounder adjustment: G-computation. |
|  |  |  |  |  |  |
| Follow-up period | Starts: at randomisation  Ends: young adulthood (age 18-30 years) | Starts: Age 17-18 years.  Ends: Age 30 years. | Starts: Age 17-18 years.  Ends: Age 27-28 years. | Starts: Age 15.5 years.  Ends: Age 29.1 years. | Starts: Age 11 years.  Ends: Age 23-29 years. |
|  |  |  |  |  |  |
| Outcome ^*^ | Elevated symptoms of common mental health problem (age 18-30 years). | Composite International Diagnostic Interview (age 21-30 years). | 21-item Depression, Anxiety, and Stress Scale (age 19-28 years). | Revised Clinical Interview Schedule, General Health Questionnaire, and Composite International Diagnostic Interview (age 20.7-29.1 years). | Kessler-10 (age 19-29 years). |
|  |  |  |  |  |  |
| Causal contrast | Comparator arm versus intervention arm: Risk Ratio. | | | | |
| Notes: ^*^ Details of measurement provided in the Measures section of the Methods; CHDS=Christchurch Health and Development Study; ATP=Australian Temperament Project; VAHCS=Victorian Adolescent Health Cohort Study; IYDS=International Youth Development Study. | | | | | |

| Table 3.2. Target Trial Emulation: What is the causal effect of adolescent mental health difficulties on subsequent young adulthood ≥monthly amphetamine use | | | | | |
| --- | --- | --- | --- | --- | --- |
| Protocol component | Target trial specification | Emulation CHDS | Emulation ATP | Emulation VAHCS | Emulation IYDS |
| Eligibility criteria | Adolescent (age 10-18 years) males and females from Australia and New Zealand across the late ‘70s into the early ‘90s (1977 to 1992). | Adolescent (age 17-18 years) males and females born in Christchurch, New Zealand in 1977. | Adolescent (age 13-18 years) males and females born in Victoria, Australia in 1983. | Adolescent (age 15-17.4 years) males and females living in Victoria, Australia as adolescents (born on average in 1978). | Adolescent (age 11-17 years) males and females living in Victoria, Australia as adolescents (born in 1988-1992). |
|  |  |  |  |  |  |
| Sub-group analyses | Male and female samples. | Male and female samples. | Male and female samples. | Male and female samples. | Male and female samples. |
|  |  |  |  |  |  |
| Treatment strategies ^*^ | Intervention arm: participants experience mental health difficulties at least once across adolescence.  Comparator arm: no intervention (no mental health difficulties). | Intervention: any mental health difficulties (Composite International Diagnostic Interview) in adolescence (age 17-18 years).  Comparator: No mental health difficulties across adolescence. | Intervention: any mental health difficulties (Short Mood and Feelings Questionnaire) in adolescence (age 13-18 years).  Comparator: No mental health difficulties across adolescence | Intervention: any mental health difficulties (Revised Clinical Interview Schedule) in adolescence (age 15.5-17.4 years).  Comparator: No mental health difficulties across adolescence | Intervention: any mental health difficulties (Short Mood and Feelings Questionnaire) in adolescence (age 11-17 years).  Comparator: No mental health difficulties across adolescence |
|  |  |  |  |  |  |
| Assignment procedures | Participants will be randomly assigned to either treatment strategy and will be aware of the strategy to which they have been assigned. | Selection of confounders.  Approach to confounder adjustment: G-computation. | Selection of confounders.  Approach to confounder adjustment: G-computation. | Selection of confounders.  Approach to confounder adjustment: G-computation. | Selection of confounders.  Approach to confounder adjustment: G-computation. |
|  |  |  |  |  |  |
| Follow-up period | Starts: At randomisation  Ends: Young adulthood (age 18-30 years) | Starts: Age 17-18 years.  Ends: Age 30 years. | Starts: Age 13-18 years.  Ends: Age 27-28 years. | Starts: Age 15.5 years.  Ends: Age 29.1 years. | Starts: Age 11 years.  Ends: Age 23-29 years. |
|  |  |  |  |  |  |
| Outcome ^*^ | Any ‘monthly’ amphetamine use (age 18-30 years). | Any ‘monthly’ amphetamine use (age 21-30 years). | Any ‘monthly’ amphetamine use (age 19-28 years). | Any ‘monthly’ amphetamine use (age 20.7-29.1 years). | Any ‘monthly’ amphetamine use (age 19-29 years). |
|  |  |  |  |  |  |
| Causal contrast | Comparator arm versus intervention arm: Risk Ratio. | | | | |
| Notes: ^*^ Details of measurement provided in the Measures section of the Methods; CHDS=Christchurch Health and Development Study; ATP=Australian Temperament Project; VAHCS=Victorian Adolescent Health Cohort Study; IYDS=International Youth Development Study. | | | | | |

Supplementary material 4. Sensitivity analyses

| Table 4.1. Developmental patterns of frequent amphetamine use and mental health difficulties across adolescence and young adulthood by cohort | | | | | | |
| --- | --- | --- | --- | --- | --- | --- |
|  | Total | | Male | | Female | |
|  | n | % (95% CI) | n | % (95% CI) | n | % (95% CI) |
|  | CHDS | | | | | |
| ≥Monthly amphetamine use |  |  |  |  |  |  |
| Adolescent | 12 | 1.09% (0.45, 1.74) | 4 | 0.84% (0.02, 1.67) | 7 | 1.34% (0.35, 2.33) |
| Young adult | 48 | 4.52% (3.22, 5.81) | 35 | 6.72% (4.49, 8.94) | 13 | 2.36% (1.05, 3.68) |
| Any mental health difficulties |  |  |  |  |  |  |
| Adolescent | 316 | 29.96% (27.16, 32.77) | 111 | 21.19% (17.63, 24.75) | 206 | 38.54% (34.35, 42.74) |
| Young adult | 529 | 50.10% (47.07, 53.14) | 202 | 38.77% (34.54, 43.00) | 327 | 61.18% (57.02, 65.34) |
| Adolescent amphetamine use and young adult mental health difficulties |  |  |  |  |  |  |
| <Monthly adolescent amphetamine use |  |  |  |  |  |  |
| No young adult mental health difficulties | 524 | 50.21% (47.16, 53.27) | 319 | 61.53% (57.28, 65.77) | 206 | 39.10% (34.91, 43.29) |
| Any young adult mental health difficulties | 520 | 49.79% (46.73, 52.84) | 199 | 38.47% (34.23, 42.72) | 321 | 60.90% (56.71, 65.09) |
| ≥Monthly adolescent amphetamine use |  |  |  |  |  |  |
| No young adult mental health difficulties | 3 | 21.09% (-4.30, 46.48) | 1 | 26.06% (-16.68, 68.79) | 1 | 17.94% (-12.74, 48.62) |
| Any young adult mental health difficulties | 9 | 78.91% (53.52, 104.30) | 3 | 73.94% (31.21, 116.68) | 6 | 82.06% (51.38, 112.74) |
| Adolescent mental health difficulties and young adult amphetamine use |  |  |  |  |  |  |
| No adolescent mental health difficulties |  |  |  |  |  |  |
| <Monthly young adult amphetamine use | 707 | 95.52% (93.98, 97.07) | 385 | 93.78% (91.35, 96.22) | 320 | 97.71% (96.05, 99.37) |
| ≥Monthly young adult amphetamine use | 33 | 4.48% (2.93, 6.02) | 26 | 6.22% (3.78, 8.65) | 8 | 2.29% (0.63, 3.95) |
| Any adolescent mental health difficulties |  |  |  |  |  |  |
| <Monthly young adult amphetamine use | 301 | 95.39% (92.99, 97.80) | 101 | 91.43% (85.91, 96.95) | 201 | 97.52% (95.37, 99.67) |
| ≥Monthly young adult amphetamine use | 15 | 4.61% (2.20, 7.01) | 10 | 8.57% (3.05, 14.09) | 5 | 2.48% (0.33, 4.63) |
|  | ATP | | | | | |
| ≥Monthly amphetamine use |  |  |  |  |  |  |
| Adolescent | 30 | 1.83% (1.11, 2.55) | 14 | 1.75% (0.68, 2.82) | 16 | 1.91% (0.87, 2.96) |
| Young adult | 177 | 10.79% (8.93, 12.65) | 99 | 12.07% (8.84, 15.30) | 78 | 9.51% (7.25, 11.76) |
| Any mental health difficulties |  |  |  |  |  |  |
| Adolescent | 637 | 38.74% (36.28, 41.20) | 228 | 27.69% (24.50, 30.89) | 409 | 49.84% (46.24, 53.45) |
| Young adult | 630 | 38.31% (35.80, 40.81) | 290 | 35.24% (31.60, 38.89) | 339 | 41.38% (37.90, 44.87) |
| Adolescent amphetamine use and young adult mental health difficulties |  |  |  |  |  |  |
| <Monthly adolescent amphetamine use |  |  |  |  |  |  |
| No young adult mental health difficulties | 1000 | 61.98% (59.43, 64.52) | 528 | 65.19% (61.51, 68.87) | 472 | 58.74% (55.21, 62.27) |
| Any young adult mental health difficulties | 614 | 38.02% (35.48, 40.57) | 282 | 34.81% (31.13, 38.49) | 332 | 41.26% (37.73, 44.79) |
| ≥Monthly adolescent amphetamine use |  |  |  |  |  |  |
| No young adult mental health difficulties | 14 | 46.46% (25.47, 67.46) | 6 | 39.68% (6.76, 72.60) | 8 | 52.13% (24.45, 79.81) |
| Any young adult mental health difficulties | 16 | 53.54% (32.54, 74.53) | 8 | 60.32% (27.40, 93.24) | 8 | 47.87% (20.19, 75.55) |
| Adolescent mental health difficulties and young adult amphetamine use |  |  |  |  |  |  |
| No adolescent mental health difficulties |  |  |  |  |  |  |
| <Monthly young adult amphetamine use | 913 | 90.70% (88.33, 93.07) | 530 | 88.91% (85.23, 92.59) | 383 | 93.29% (90.48, 96.11) |
| ≥Monthly young adult amphetamine use | 94 | 9.30% (6.93, 11.67) | 66 | 11.09% (7.41, 14.77) | 28 | 6.71% (3.89, 9.52) |
| Any adolescent mental health difficulties |  |  |  |  |  |  |
| <Monthly young adult amphetamine use | 553 | 86.85% (83.82, 89.87) | 195 | 85.37% (79.34, 91.40) | 359 | 87.67% (84.17, 91.18) |
| ≥Monthly young adult amphetamine use | 84 | 13.15% (10.13, 16.18) | 33 | 14.63% (8.60, 20.66) | 50 | 12.33% (8.82, 15.83) |
|  | VAHCS | | | | | |
| ≥Monthly amphetamine use |  |  |  |  |  |  |
| Adolescent | 39 | 1.99% (1.34, 2.64) | 20 | 2.12% (1.16, 3.09) | 19 | 1.86% (0.99, 2.72) |
| Young adult | 137 | 7.06% (5.72, 8.40) | 85 | 8.99% (6.74, 11.24) | 52 | 5.25% (3.65, 6.84) |
| Any mental health difficulties |  |  |  |  |  |  |
| Adolescent | 768 | 39.56% (37.38, 41.74) | 251 | 26.68% (23.84, 29.51) | 517 | 51.70% (48.60, 54.81) |
| Young adult | 723 | 37.27% (34.98, 39.56) | 255 | 27.08% (24.04, 30.12) | 468 | 46.87% (43.63, 50.11) |
| Adolescent amphetamine use and young adult mental health difficulties |  |  |  |  |  |  |
| <Monthly adolescent amphetamine use |  |  |  |  |  |  |
| No young adult mental health difficulties | 1199 | 63.09% (60.77, 65.41) | 674 | 73.23% (70.17, 76.29) | 525 | 53.57% (50.29, 56.85) |
| Any young adult mental health difficulties | 702 | 36.91% (34.59, 39.23) | 247 | 26.77% (23.71, 29.83) | 455 | 46.43% (43.15, 49.71) |
| ≥Monthly adolescent amphetamine use |  |  |  |  |  |  |
| No young adult mental health difficulties | 17 | 44.85% (26.95, 62.75) | 12 | 58.51% (32.88, 84.14) | 6 | 30.15% (8.23, 52.07) |
| Any young adult mental health difficulties | 22 | 55.15% (37.25, 73.05) | 8 | 41.49% (15.86, 67.12) | 13 | 69.85% (47.93, 91.77) |
| Adolescent mental health difficulties and young adult amphetamine use |  |  |  |  |  |  |
| No adolescent mental health difficulties |  |  |  |  |  |  |
| <Monthly young adult amphetamine use | 1100 | 93.90% (92.24, 95.55) | 634 | 91.92% (89.39, 94.44) | 466 | 96.73% (94.82, 98.63) |
| ≥Monthly young adult amphetamine use | 72 | 6.10% (4.45, 7.76) | 56 | 8.08% (5.56, 10.61) | 16 | 3.27% (1.37, 5.18) |
| Any adolescent mental health difficulties |  |  |  |  |  |  |
| <Monthly young adult amphetamine use | 703 | 91.47% (89.23, 93.72) | 222 | 88.51% (83.83, 93.19) | 480 | 92.91% (90.45, 95.37) |
| ≥Monthly young adult amphetamine use | 65 | 8.53% (6.28, 10.77) | 29 | 11.49% (6.81, 16.17) | 37 | 7.09% (4.63, 9.55) |
|  | IYDS | | | | | |
| ≥Monthly amphetamine use |  |  |  |  |  |  |
| Adolescent | 68 | 2.37% (1.81, 2.94) | 31 | 2.20% (1.41, 2.99) | 38 | 2.53% (1.72, 3.34) |
| Young adult | 395 | 13.69% (12.32, 15.07) | 244 | 17.52% (15.31, 19.74) | 150 | 10.11% (8.49, 11.73) |
| Any mental health difficulties |  |  |  |  |  |  |
| Adolescent | 1334 | 46.26% (44.44, 48.08) | 499 | 35.79% (33.27, 38.31) | 835 | 56.08% (53.56, 58.60) |
| Young adult | 1675 | 58.07% (56.17, 59.98) | 729 | 52.28% (49.46, 55.10) | 945 | 63.50% (60.96, 66.04) |
| Adolescent amphetamine use and young adult mental health difficulties |  |  |  |  |  |  |
| <Monthly adolescent amphetamine use |  |  |  |  |  |  |
| No young adult mental health difficulties | 1193 | 42.37% (40.44, 44.31) | 656 | 48.12% (45.26, 50.97) | 537 | 36.98% (34.38, 39.57) |
| Any young adult mental health difficulties | 1623 | 57.63% (55.69, 59.56) | 708 | 51.88% (49.03, 54.74) | 914 | 63.02% (60.43, 65.62) |
| ≥Monthly adolescent amphetamine use |  |  |  |  |  |  |
| No young adult mental health difficulties | 16 | 23.47% (12.09, 34.85) | 9 | 29.99% (11.23, 48.75) | 7 | 18.16% (5.04, 31.27) |
| Any young adult mental health difficulties | 52 | 76.53% (65.15, 87.91) | 22 | 70.01% (51.25, 88.77) | 31 | 81.84% (68.73, 94.96) |
| Adolescent mental health difficulties and young adult amphetamine use |  |  |  |  |  |  |
| No adolescent mental health difficulties |  |  |  |  |  |  |
| <Monthly young adult amphetamine use | 1352 | 87.25% (85.41, 89.10) | 748 | 83.51% (80.76, 86.26) | 604 | 92.38% (90.22, 94.54) |
| ≥Monthly young adult amphetamine use | 198 | 12.75% (10.90, 14.59) | 148 | 16.49% (13.74, 19.24) | 50 | 7.62% (5.46, 9.78) |
| Any adolescent mental health difficulties |  |  |  |  |  |  |
| <Monthly young adult amphetamine use | 1137 | 85.21% (83.15, 87.26) | 402 | 80.62% (76.78, 84.46) | 734 | 87.94% (85.63, 90.26) |
| ≥Monthly young adult amphetamine use | 197 | 14.79% (12.74, 16.85) | 97 | 19.38% (15.54, 23.22) | 101 | 12.06% (9.74, 14.37) |
| Notes: CHDS=Christchurch Health and Development Study; ATP=Australian Temperament Project; VAHCS=Victorian Adolescent Health Cohort Study; IYDS=International Youth and Development Study. | | | | | | |

| Table 4.2. By study associations between amphetamine use and mental health difficulties across subsequent developmental periods | | | | | | |
| --- | --- | --- | --- | --- | --- | --- |
|  | No  adjustment ^a^ | | Potential confounding  factor adjustment ^b^ | | Adolescent outcome  adjustment ^c^ | |
|  | RR | 95% CI | RR | 95% CI | RR | 95% CI |
|  | Total | | | | | |
| Outcome:  Young adult mental health difficulties |  |  |  |  |  |  |
| Adolescent amphetamine use |  |  |  |  |  |  |
| <Monthly | (base) |  | (base) |  | (base) |  |
| CHDS ≥Monthly | 1.58 | (1.13, 2.21) | 1.45 | (0.93, 2.27) | 1.39 | (0.91, 2.14) |
| ATP ≥Monthly | 1.40 | (0.93, 2.10) | 1.38 | (0.90, 2.11) | 1.27 | (0.84, 1.90) |
| VAHCS ≥Monthly | 1.49 | (1.07, 2.08) | 1.26 | (0.85, 1.85) | 1.17 | (0.80, 1.72) |
| IYDS ≥Monthly | 1.33 | (1.14, 1.55) | 1.19 | (1.00, 1.42) | 1.20 | (1.01, 1.43) |
| Outcome:  Young adult ≥monthly amphetamine use |  |  |  |  |  |  |
| Adolescent mental health difficulties |  |  |  |  |  |  |
| None | (base) |  | (base) |  | (base) |  |
| CHDS any | 1.03 | (0.55, 1.93) | 1.05 | (0.53, 2.10) | 1.02 | (0.51, 2.03) |
| ATP any | 1.42 | (1.00, 2.00) | 1.45 | (1.01, 2.09) | 1.37 | (0.95, 1.98) |
| VAHCS any | 1.40 | (0.96, 2.04) | 1.26 | (0.80, 1.97) | 1.21 | (0.77, 1.89) |
| IYDS any | 1.16 | (0.95, 1.42) | 1.07 | (0.85, 1.33) | 1.07 | (0.86, 1.34) |
|  | Male | | | | | |
| Outcome:  Young adult mental health difficulties |  |  |  |  |  |  |
| Adolescent amphetamine use |  |  |  |  |  |  |
| <Monthly | (base) |  | (base) |  | (base) |  |
| CHDS ≥Monthly | 1.92 | (1.05, 3.49) | 1.74 | (0.75, 4.01) | 1.68 | (0.79, 3.56) |
| ATP ≥Monthly | 1.71 | (0.97, 3.02) | 1.80 | (0.96, 3.39) | 1.70 | (0.92, 3.13) |
| VAHCS ≥Monthly | 1.53 | (0.80, 2.92) | 1.23 | (0.61, 2.46) | 1.10 | (0.56, 2.18) |
| IYDS ≥Monthly | 1.35 | (1.02, 1.78) | 1.19 | (0.88, 1.60) | 1.23 | (0.91, 1.65) |
| Outcome:  Young adult ≥monthly amphetamine use |  |  |  |  |  |  |
| Adolescent mental health difficulties |  |  |  |  |  |  |
| None |  |  |  |  |  |  |
| CHDS any | 1.37 | (0.64, 2.95) | 1.29 | (0.60, 2.80) | 1.31 | (0.61, 2.82) |
| ATP any | 1.32 | (0.78, 2.22) | 1.16 | (0.68, 1.97) | 1.11 | (0.65, 1.90) |
| VAHCS any | 1.42 | (0.85, 2.37) | 1.10 | (0.64, 1.90) | 1.05 | (0.61, 1.81) |
| IYDS any | 1.17 | (0.90, 1.53) | 0.95 | (0.73, 1.23) | 0.96 | (0.74, 1.25) |
|  | Female | | | | | |
| Outcome:  Young adult mental health difficulties |  |  |  |  |  |  |
| Adolescent amphetamine use |  |  |  |  |  |  |
| <Monthly | (base) |  | (base) |  | (base) |  |
| CHDS ≥Monthly | 1.34 | (0.91, 1.99) | 1.25 | (0.78, 2.00) | 1.20 | (0.74, 1.94) |
| ATP ≥Monthly | 1.15 | (0.63, 2.09) | 1.16 | (0.63, 2.14) | 1.02 | (0.57, 1.80) |
| VAHCS ≥Monthly | 1.50 | (1.09, 2.08) | 1.22 | (0.84, 1.76) | 1.18 | (0.82, 1.69) |
| IYDS ≥Monthly | 1.30 | (1.10, 1.53) | 1.20 | (0.99, 1.45) | 1.18 | (0.98, 1.43) |
| Outcome:  Young adult ≥monthly amphetamine use |  |  |  |  |  |  |
| Adolescent mental health difficulties |  |  |  |  |  |  |
| None | (base) |  | (base) |  | (base) |  |
| CHDS any | 1.08 | (0.35, 3.34) | 1.17 | (0.36, 3.85) | 1.10 | (0.33, 3.67) |
| ATP any | 1.84 | (1.11, 3.06) | 1.86 | (1.10, 3.13) | 1.69 | (1.00, 2.86) |
| VAHCS any | 2.19 | (1.12, 4.27) | 1.48 | (0.73, 2.99) | 1.44 | (0.72, 2.91) |
| IYDS any | 1.58 | (1.13, 2.23) | 1.17 | (0.82, 1.66) | 1.17 | (0.82, 1.67) |
| Notes: ^a^ Includes no adjustment; ^b^ includes adjustment for cohort and potential confounding factors excluding adolescent outcome; ^c^ includes adjustment for cohort and potential confounding factors including adolescent outcome; Models include interactions between exposure, cohort, and participant sex when variables are present in the model; CHDS=Christchurch Health and Development Study; ATP=Australian Temperament Project; VAHCS=Victorian Adolescent Health Cohort Study; IYDS=International Youth and Development Study. | | | | | | |

| Table 4.3. Associations between frequent amphetamine use and mental health difficulties across subsequent developmental periods in the harmonised data, *excluding participants who reported any but less than monthly amphetamine use* | | | | | | |
| --- | --- | --- | --- | --- | --- | --- |
|  | Cohort  adjustment ^a^ | | Potential confounding  factor adjustment ^b^ | | Adolescent outcome  adjustment ^c^ | |
|  | RR | 95% CI | RR | RR | 95% CI | RR |
|  | Total (N=6,753) | | | | | |
| Outcome:  Young adult mental health difficulties |  |  |  |  |  |  |
| Adolescent amphetamine use |  |  |  |  |  |  |
| <Monthly | (base) |  | (base) |  | (base) |  |
| ≥Monthly | 1.40 | (1.19, 1.63) | 1.27 | (1.07, 1.50) | 1.23 | (1.04, 1.45) |
| Outcome:  Young adult ≥monthly amphetamine use |  |  |  |  |  |  |
| Adolescent mental health difficulties |  |  |  |  |  |  |
| None | (base) |  | (base) |  | (base) |  |
| Any | 1.26 | (1.06, 1.50) | 1.24 | (1.02, 1.50) | 1.22 | (1.01, 1.48) |
|  | Male (N=3,248) | | | | | |
| Outcome:  Young adult mental health difficulties |  |  |  |  |  |  |
| Adolescent amphetamine use |  |  |  |  |  |  |
| <Monthly | (base) |  | (base) |  | (base) |  |
| ≥Monthly | 1.50 | (1.16, 1.94) | 1.25 | (0.94, 1.67) | 1.22 | (0.93, 1.60) |
| Outcome:  Young adult ≥monthly amphetamine use |  |  |  |  |  |  |
| Adolescent mental health difficulties |  |  |  |  |  |  |
| None | (base) |  | (base) |  | (base) |  |
| Any | 1.33 | (1.06, 1.68) | 1.16 | (0.92, 1.47) | 1.15 | (0.91, 1.45) |
|  | Female (N=3,505) | | | | | |
| Outcome:  Young adult mental health difficulties |  |  |  |  |  |  |
| Adolescent amphetamine use |  |  |  |  |  |  |
| <Monthly | (base) |  | (base) |  | (base) |  |
| ≥Monthly | 1.36 | (1.14, 1.63) | 1.27 | (1.05, 1.54) | 1.23 | (1.02, 1.48) |
| Outcome:  Young adult ≥monthly amphetamine use |  |  |  |  |  |  |
| Adolescent mental health difficulties |  |  |  |  |  |  |
| None | (base) |  | (base) |  | (base) |  |
| Any | 1.67 | (1.24, 2.24) | 1.33 | (0.98, 1.81) | 1.31 | (0.97, 1.77) |
| Notes: ^a^ Includes adjustment for cohort; ^b^ includes adjustment for cohort and potential confounding factors excluding adolescent outcome; ^c^ includes adjustment for cohort and potential confounding factors including adolescent outcome; Models include interactions between exposure, cohort, and participant sex when variables are present in the model. | | | | | | |

| Table 4.4. Associations between frequent amphetamine use and mental health difficulties across subsequent developmental periods in the harmonised data, *using non-imputed data* | | | | | | |
| --- | --- | --- | --- | --- | --- | --- |
|  | Cohort  adjustment ^a^ | | Potential confounding  factor adjustment ^b^ | | Adolescent outcome  adjustment ^c^ | |
|  | RR | 95% CI | RR | RR | 95% CI | RR |
|  | Total (N=4,334 to 6,265) | | | | | |
| Outcome:  Young adult mental health difficulties |  |  |  |  |  |  |
| Adolescent amphetamine use |  |  |  |  |  |  |
| <Monthly | (base) |  | (base) |  | (base) |  |
| ≥Monthly | 1.47 | (1.29, 1.67) | 1.40 | (1.20, 1.63) | 1.30 | (1.12, 1.51) |
| Outcome:  Young adult ≥monthly amphetamine use |  |  |  |  |  |  |
| Adolescent mental health difficulties |  |  |  |  |  |  |
| None | (base) |  | (base) |  | (base) |  |
| Any | 1.28 | (1.07, 1.54) | 1.24 | (0.98, 1.58) | 1.21 | (0.94, 1.54) |
|  | Male (N=1,925 to 2,897) | | | | | |
| Outcome:  Young adult mental health difficulties |  |  |  |  |  |  |
| Adolescent amphetamine use |  |  |  |  |  |  |
| <Monthly | (base) |  | (base) |  | (base) |  |
| ≥Monthly | 1.64 | (1.31, 2.06) | 1.37 | (1.02, 1.83) | 1.30 | (0.97, 1.73) |
| Outcome:  Young adult ≥monthly amphetamine use |  |  |  |  |  |  |
| Adolescent mental health difficulties |  |  |  |  |  |  |
| None | (base) |  | (base) |  | (base) |  |
| Any | 1.31 | (1.03, 1.67) | 1.06 | (0.80, 1.40) | 1.06 | (0.79, 1.41) |
|  | Female (N=2,409 to 3,368) | | | | | |
| Outcome:  Young adult mental health difficulties |  |  |  |  |  |  |
| Adolescent amphetamine use |  |  |  |  |  |  |
| <Monthly | (base) |  | (base) |  | (base) |  |
| ≥Monthly | 1.34 | (1.15, 1.58) | 1.40 | (1.21, 1.62) | 1.27 | (1.10, 1.45) |
| Outcome:  Young adult ≥monthly amphetamine use |  |  |  |  |  |  |
| Adolescent mental health difficulties |  |  |  |  |  |  |
| None | (base) |  | (base) |  | (base) |  |
| Any | 1.71 | (1.27, 2.32) | 1.44 | (0.99, 2.09) | 1.37 | (0.93, 2.00) |
| Notes: ^a^ Includes adjustment for cohort; ^b^ includes adjustment for cohort and potential confounding factors excluding adolescent outcome; ^c^ includes adjustment for cohort and potential confounding factors including adolescent outcome; Models include interactions between exposure, cohort, and participant sex when variables are present in the model. | | | | | | |

Supplementary material references

1. World Health Organization. Composite International Diagnostic Interview. Geneva, Switzerland; 1993.

2. Turner N, Joinson C, Peters TJ, Wiles N, Lewis G. Validity of the Short Mood and feelings questionnaire in late adolescence. Psychol Assess. 2014;26(3):752–62.

3. Angold A, Costello EJ, Messer S, Pickles A, Winder F, D S. The development of a short questionnaire for use in epidemiological studies of depression in children and adolescents. Int J Methods Psychiatr Res. 1995;5:237–49.

4. Thapar A, McGuffin P. Validity of the shortened Mood and Feelings Questionnaire in a community sample of children and adolescents: A preliminary research note. Psychiatry Res. 1998;81(2):259–68.

5. Lovibond SH, Lovibond PF. Manual for the Depression Anxiety Stress Scales. Psychology Foundation of Australia. 1995.

6. Lewis G, Pelosi AJ, Araya R, Dunn G. Measuring psychiatric disorder in the community: A standardized assessment for use by lay interviewers. Psychol Med. 1992;22(2):465–86.

7. Donath S. The Validity of the 12-Item General Health Questionnaire in Australia: A Comparison Between Three Scoring Methods. Australian & New Zealand Journal of Psychiatry [Internet]. 2001 Apr 17;35(2):231–5. Available from: http://journals.sagepub.com/doi/10.1046/j.1440-1614.2001.00869.x

8. Goldberg DP, Gater R, Sartorius N, Ustun TB, Piccinelli M, Gureje O, et al. The validity of two versions of the GHQ in the WHO study of mental illness in general health care. Psychol Med. 1997 Jan;27(1):191–7.

9. World Health Organization. Composite International Diagnostic Interview, CIDI-auto 2.1: administrator’s guide and reference. Geneva; 1997.

10. Kessler RC, Andrews G, Mroczek D, Ustun B, Wittchen H. The World Health Organization Composite International Diagnostic Interview short‐form (CIDI‐SF). Int J Methods Psychiatr Res. 1998 Nov;7(4):171–85.

11. Kessler RC, Barker PR, Colpe LJ, Epstein JF, Gfroerer JC, Hiripi E, et al. Screening for Serious Mental Illness in the General Population. Arch Gen Psychiatry [Internet]. 2003 Feb 1;60(2):184. Available from: http://archpsyc.jamanetwork.com/article.aspx?doi=10.1001/archpsyc.60.2.184

12. Andrews G, Slade T. Interpreting scores on the Kessler Psychological Distress Scale (K10). Aust N Z J Public Health [Internet]. 2001 Dec;25(6):494–7. Available from: https://linkinghub.elsevier.com/retrieve/pii/S1326020023036105

13. Cairns RB, Cairns BD. Predicting Aggressive Patterns in Girls and Boys: A Developmental Study. Aggress Behav [Internet]. 10:227–42. Available from: https://onlinelibrary.wiley.com/terms-and-conditions

14. Gresham F, Elliott S. Manual for the Social Skills Rating System. Circle Pines MN: American Guidance Service; 1990.

15. Fergusson DM, Horwood LJ, Lynskey MT. The Effects of Conduct Disorder and Attention Deficit in Middle Childhood on Offending and Scholastic Ability at Age 13. Journal of Child Psychology and Psychiatry. 1993;34(6):899–916.

16. Rutter M, Tizard J, Whitmore K. Education, Health and Behaviour. London: Longman; 1970.

17. Jackson DN. Personality research form manual. 2nd ed. Port Huron, MI: Research Psychologists Press; 1974.

18. Quay HC, Peterson DR. Manual For the Revised Behaviour Problem Checklist. Odessa, FL: Psychological Assessment Resources; 1987.

19. Fergusson DM, Horwood LJ, Nagin DS. Offending trajectories in a New Zealand birth cohort. Criminology. 2000;38(2):525–52.

20. Moffitt TE, Silva PA. Self-reported delinquency: Results from an instrument for new zealand. Australian & New Zealand Journal of Criminology. 1988;21(4):227–40.

21. Hemphill SA, McMorris BJ, Toumbourou JW, Herrenkohl TI, Catalano RF, Mathers M. Rates of student-reported antisocial behavior, school suspensions, and arrests in Victoria, Australia and Washington State, United States. Journal of School Health. 2007 Aug;77(6):303–11.

22. Armsden GC, Greenberg MT. The inventory of parent and peer attachment: Individual differences and their relationship to psychological well-being in adolescence. J Youth Adolesc [Internet]. 1987 Oct;16(5):427–54. Available from: http://www.ncbi.nlm.nih.gov/pubmed/24277469

23. Jenkins R, Mann AH, Belsey E. The background, design and use of a short interview to assess social stress and support in research and clinical settings. Soc Sci Med E [Internet]. 1981 Aug;15(3):195–203. Available from: https://linkinghub.elsevier.com/retrieve/pii/0271538481900132

24. Glaser RR, Horn ML Van, Arthur MW, Hawkins JD, Catalano RF. Measurement properties of the Communities That Care® Youth survey across demographic groups. Vol. 21, Journal of Quantitative Criminology. 2005. p. 73–102.

25. Arthur MW, Hawkins JD, Pollard JA, Catalano RF, Baglioni Jr. AJ. Measuring Risk And Protective Factors For Substance Use, Delinquency, And Other Adolescent Problem Behaviors: The Communities That Care Youth Survey. Eval Rev. 2002 Dec 1;26(6):575–601.
